# Effects of the CK2 Inhibitors CX-4945 and CX-5011 on Drug-Resistant Cells

**DOI:** 10.1371/journal.pone.0049193

**Published:** 2012-11-08

**Authors:** Sofia Zanin, Christian Borgo, Cristina Girardi, Sean E. O'Brien, Yoshihiko Miyata, Lorenzo A. Pinna, Arianna Donella-Deana, Maria Ruzzene

**Affiliations:** 1 Department of Biomedical Sciences and National Research Council Institute of Neurosciences, University of Padova, Padova, Italy; 2 Cylene Pharmaceuticals, San Diego, California, United States of America; 3 Department of Cell and Developmental Biology, Graduate School of Biostudies, Kyoto University, Kyoto, Japan; 4 Venetian Institute of Molecular Medicine, Padova, Italy; Cedars-Sinai Medical Center, United States of America

## Abstract

CK2 is a pleiotropic protein kinase, which regulates many survival pathways and plays a global anti-apoptotic function. It is highly expressed in tumor cells, and is presently considered a promising therapeutic target. Among the many inhibitors available for this kinase, the recently developed CX-4945 and CX-5011 have proved to be very potent, selective and effective in inducing cell death in tumor cells; CX-4945 has recently entered clinical trials. However, no data are available on the efficacy of these compounds to overcome drug resistance, a major reasons of cancer therapy failure. Here we address this point, by studying their effects in several tumor cell lines, each available as variant R resistant to drug-induced apoptosis, and normal-sensitive variant S. We found that the inhibition of endogenous CK2 was very similar in S and R treated cells, with more than 50% CK2 activity reduction at sub-micromolar concentrations of CX-4945 and CX-5011. A consequent apoptotic response was induced both in S and R variants of each pairs. Moreover, the combined treatment of CX-4945 plus vinblastine was able to sensitize to vinblastine R cells that are otherwise almost insensitive to this conventional antitumor drug. Consistently, doxorubicin accumulation in multidrug resistant (MDR) cells was greatly increased by CX-4945.

In summary, we demonstrated that all the R variants are sensitive to CX-4945 and CX-5011; since some of the treated R lines express the extrusion pump Pgp, often responsible of the MDR phenotype, we can also conclude that the two inhibitors can successfully overcome the MDR phenomenon.

## Introduction

CK2 is a Ser/Thr protein kinase usually present in the cells as a tetrameric enzyme composed of two catalytic (α and/or α') and two regulatory (β) subunits. It is constitutively active and ubiquitously expressed, and phosphorylates such a striking number of substrates to be considered the most pleiotropic protein kinase [1]. It is involved in several cellular processes, such as cell cycle, gene expression, protein synthesis, signal transduction and metabolism; however, its hall-mark is considered its prosurvival and anti-apoptotic function [2]–[5]. This is supported by the observation that many CK2 substrates are proteins involved in cell death/survival, and, more importantly, that the reduction of CK2 activity or expression (induced by cell treatment with specific inhibitors or by RNA interference technology, respectively) is invariantly followed by cell death, mainly due to apoptosis (reviewed in [6]).

Consistent with the anti-apoptotic function of CK2, cancer cells, which are characterized by rapid proliferation and defective apoptosis, express particularly high levels of CK2. It has a special role in tumorigenesis [7], potentiating pathways that are frequently up-regulated or untimely activated in cancer [8], and it has consequently been defined as “a key player in cancer biology” [9]. Whenever comparison has been performed, CK2 has been shown significantly more abundant in tumor cells than in healthy counterparts. However, at the same time tumors rely more on CK2 for their survival, and this phenomenon, described as “addiction” to CK2 of cancer cells [6], explains why they are more sensitive to its inhibition or knocking-down, compared to normal cells.

On these bases, CK2 is presently considered a promising therapeutic target [7], [10], also exploiting the fact that, due to the peculiar structure of the CK2 catalytic site [11], [12], several very specific inhibitors are available (reviewed in [13]). Many of them have already proved to be able to kill cancer cells and in some cases also employed for successful animal treatment (e.g. [14]–[18]).

The two compounds CX-4945 and CX-5011 are among the most selective and effective CK2 inhibitors developed so far. They are tricyclic ATP-competitive compounds, displaying a Ki in vitro <1 nM [17], [19], and an unprecedented selectivity for CK2, proved by profiling them against a panel of 235 protein kinases [19]. Both CX-4945 and CX-5011 are able to cause apoptosis in a number of cancer cell lines and are effective in reducing tumor size in animal models of cancer [17], [20]; CX-4945 is orally bio-available, and is presently in clinical trial for treatment of different kinds of cancer [17]. However, CX-4945 and CX-5011 have never been tested in cells that are resistant to drug-induced apoptosis.

Apoptosis resistance is a major reason of cancer therapy failure; its mechanisms can be different and multifaceted, and is only partially understood. In many cases it is due to the (over)expression of extrusion pumps of the ABC-transporter family, such as Pgp, which drive drugs outside the cell and reduce their effective concentration [21]. Cells expressing these pumps are selected for their survival in response to treatment with a certain drug, but usually a cross-resistance occurs towards other compounds, even not structurally related; in these cases, cells are indicated as multidrug-resistant (MDR). Many other mechanisms have been reported to be involved in apoptosis resistance, including alteration in genetic features, DNA repair, drug target molecules, metabolic and growth pathways [22], [23]. In some cases, specific resistance is observed, such as that towards Imatinib and its derivatives targeting Bcr-Abl tyrosine kinase, frequently due to kinase mutations, but also to epigenetic changes, alternative splicing or induction of compensatory signaling pathways [24].

CK2 has been already associated to the phenomenon of drug resistance: it phosphorylates Pgp [25] and another extrusion pump, MRP1 [26] and its inhibition allows a higher accumulation of drugs in Pgp [27] or MRP1 [26] expressing cells, suggesting that CK2 can up-regulate the Pgp function. Moreover, we have previously found that the CK2 catalytic subunit is overexpressed in a MDR cell line compared to the non-MDR counterpart, and that its overexpression contributes to the maintenance of the resistant phenotype [27].

Here we evaluate the efficacy of the CK2 inhibitors CX-4945 and CX-5011 in a number of different cell lines, available as pairs, each pair containing a variant selected for resistance to drug-induced apoptosis, and we demonstrate that these compounds can overcome the problem of drug resistance.

## Materials and Methods

### Antibodies

CK2α-subunit antisera were raised in rabbit against the sequence of the human protein at C-terminus [376–391], total Akt and total Cdc37 antibodies were from Santa Cruz Biotechnology, β-actin monoclonal antibodies were from Sigma, PARP antibodies were from Roche, Pgp monoclonal antibodies were from Calbiochem, Akt Sp129 phospho-specific antibodies were raised in rabbit and purified as elsewhere described [28]; Cdc37 Sp13 phospho-specific antibodies were from Abcam; secondary antibodies towards rabbit and mouse IgG, conjugated to horse radish peroxidase, were from PerkinElmer.

### Cell lines

The following cell lines were used in this study: human T lymphoblastoid CEM cells (normal sensitive, S-CEM, and their MDR variant, R-CEM, selection with 0.1 µg/ml vinblastine, Vbl [27]); human osteosarcoma U2OS cells (normal sensitive, S-U2OS, and their MDR variant, R-U2OS [29]); human ovarian carcinoma 2008 cells (normal sensitive, S-2008, and their cisplatin-resistant variant, R-2008 [30]); chronic myeloid leukemia (CML) LAMA84 [31], KCL22 [32], and K562 [32] cell lines, sensitive or resistant to Imatinib, kindly supplied by Dr. C. Gambacorti-Passerini.

### Inhibitors

CX-4945 (5-(3-chlorophenylamino)benzo[c][2,6]naphthyridine-8-carboxyliuc acid) and CX-5011 (5-(3-ethynylphenylamino)pyrimido[4,5-c]quinoline-8-carboxylic acid) were synthetized by Cylene Pharmaceutical. Solutions were made in dimethylsulfoxide (DMSO).

### Cell culture and treatment

Cells were cultured in an atmosphere containing 5% CO_2_; CEM, LAMA84, K562 and KCL22 cell lines were maintained in RPMI 1640 medium (Sigma), U2OS and 2008 lines were maintained in D-MEM medium (Sigma); both media were supplemented with 10% (v/v) fetal calf serum (FCS), 2 mM L-glutamine, 100 U/ml penicillin, and 100 µg/ml streptomycin. Cell treatments with inhibitors were performed in the culture medium, but with 1% FCS, where not differently indicated. Control cells were treated with equal amounts of the inhibitor solvent. At the end of the incubations, cells were harvested by centrifugation, washed, and lysed as indicated below.

### Cell viability

Cell viability was detected by means of MTT (3-(4,5-dimethylthiazol-2-yl)-3,5-diphenyltriazolium bromide) reagent: cells (10^5^ cells/100 µl) were incubated for variable times in a 96-well plate under the indicated conditions. 1 h before the end of the incubation, 10 µl of MTT solution (5 mg/ml in PBS) were added to each well. Incubations were stopped by addition of 20 µl of lysis solution at pH 4.7, as described elsewhere [33]. Plates were read for OD at λ590 nm, in a Titertek Multiskan Plus plate reader (Flow Laboratories). DC_50_ (concentrations inducing 50% of cell death) values were calculated with Prism 4.0c software (GraphPad Software).

### Apoptosis assay

Apoptosis was evaluated by means of the Cell Detection Elisa kit (Roche), based on the quantification of nucleosomes present in the cytosol of the apoptotic cells, by measuring the absorbance at λ405–λ490, following the manufacturer's instructions. About 10 000 cells were used for each determination.

### Cell lysis and western blot analysis

For lysate preparation, cells were lysed as described in [27]. Protein concentration was determined by the Bradford method. Equal amounts of proteins were loaded on 11% SDS-PAGE, blotted on Immobilon-P membranes (Millipore), and processed in western blot (WB) with the indicated antibody, detected by chemiluminescence. Quantitation of the signal was obtained by chemiluminescence detection on a Kodak Image Station 440MM PRO and analysis with the Kodak 1D Image software.

### CK2 activity assay

1–2 µg of lysate proteins were incubated for 10 min at 30°C with 0.1 mM CK2-specific peptide RRRADDSDDDDD, in the presence of phosphorylation reaction mixture [34]. Phosphorylation of endogenous proteins was performed on 5 µg of lysate proteins under the same conditions, but without the addition of the peptide substrate, and analyzed by SDS/PAGE and digital autoradiography (CyclonePlus, PerkinElmer).

### Combined treatments

Drug interactions were assessed by treating cells with increasing concentrations of both Vbl and CX-4945, at fixed concentration ratio, as indicated. The combination index (CI) [35], was calculated with the software Calcusyn (Biosoft); CI<1, CI = 1, and CI>1 indicate synergistic, additive and antagonistic effects, respectively. Values are reported as mean of three separate experiments ± SE.

### Doxorubicin accumulation measurement

Doxorubicin measurements were performed as previously described [27]. Briefly, CEM cells were treated with 25 µM doxorubicin (Sigma) for 30 min, after 30 min preincubation in the absence or in the presence of CX-4945. After washing with phosphate-buffered saline, cells were lysed in the same buffer, supplemented with 1% (w/v) SDS. Doxorubicin content was determined fluorimetrically (λexc.485 nm, λem.590 nm).

### Statistical analysis

All experiments were performed at least 3 times, each time in duplicate; graphs and statistical analysis were performed with Prism 4.0c software (GraphPad Software).

## Results

**Figure 1:**
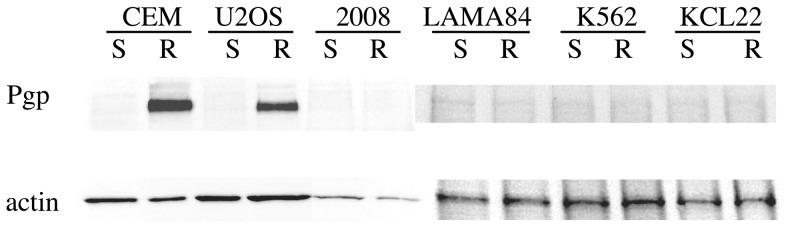
Pgp expression. 10 µg of proteins from total lysates of the indicated cells were loaded onto an SDS-PAGE, blotted and analyzed by WB with anti-Pgp or anti-actin.

For this study, we used 6 different pairs of cell lines, each available in two variants: the S variant, corresponding to cells normally sensitive to drug-induced apoptosis, and the R variant, characterized by drug resistance due to different reasons. As shown in Figure 1, CEM and U2OS resistant cells express the Pgp pump, responsible for the extrusion of a variety of drugs from the cell (reviewed by Goda et al., [36]); thus R-CEM and R-U2OS are considered MDR (multi-drug resistant) lines. Pgp is not appreciably expressed by the R variants of the other cell lines, which instead display resistance for specific drugs: 2008 cells were selected for their resistance to cisplatinum, while R-LAMA84, R-KCL22 and R-K562 are resistant to Imatinib [37].

**Figure 2:**
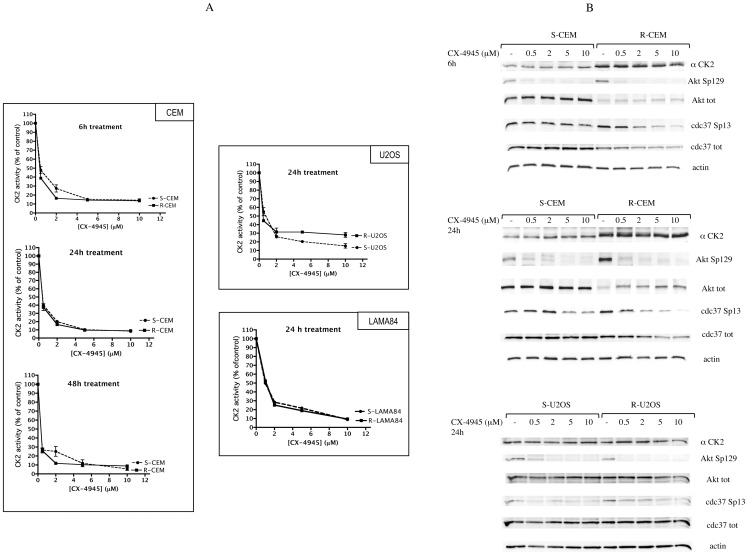
CK2 activity in CX-4945-treated cells. (A) CK2 activity was measured towards a synthetic specific peptide. 1–2 µg of proteins from total lysates of the indicated cells were incubated with the peptide and a radioactive phosphorylation mixture (see Materials and Methods). Activity is reported as percentage of that found in vehicle-treated control cells. Mean ± SE values of four independent experiments are shown. (B) 10 µg of total proteins were analyzed with the indicated antibodies; actin was used to normalize the loading. Representative WB of three independent experiments are shown.

**Figure 3:**
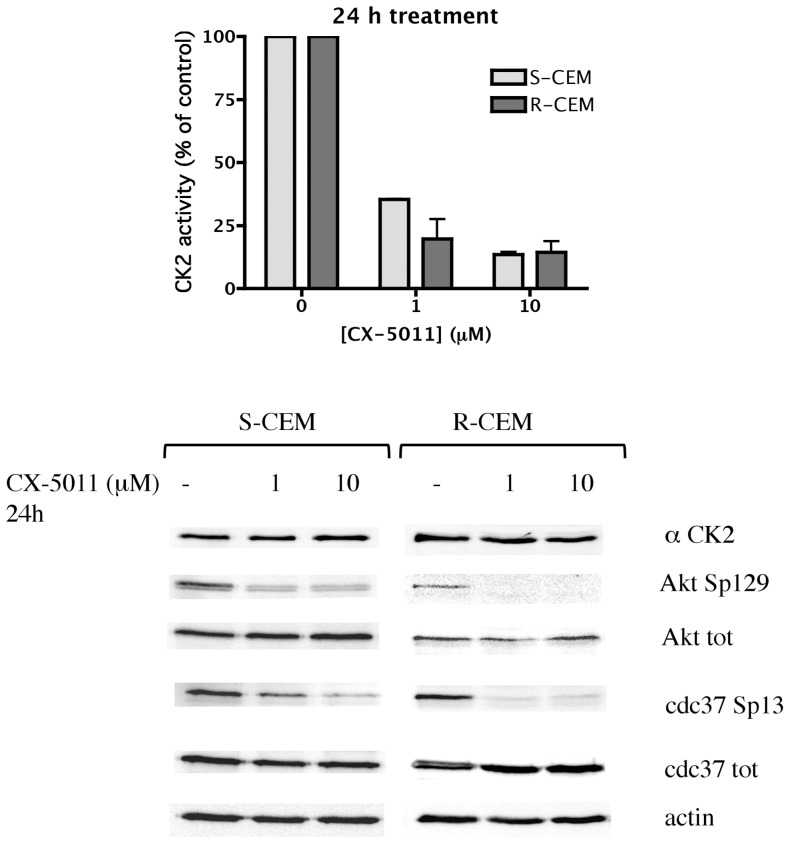
CK2 activity in CX-5011-treated cells. S-CEM and R-CEM cells were treated for 24 h with different concentrations of CX-5011. Cell lysates were analyzed for the activity of CK2 towards a synthetic specific peptide (upper graph), or by WB with the indicated antibodies (lower panel, separate development for S- and R-CEM cells). See details in the legend of Figure 2.

We first measured CK2 activity in cells treated for different times with increasing concentrations of the compounds. To this purpose we exploited two strategies: in vitro assays of endogenous CK2 present in total lysates from treated cells [34], and evaluation of the phosphorylation state of CK2 specific intracellular substrates, by means of phospho-specific antibodies towards two key targets of CK2, namely Akt S129 [28] and Cdc37 S13 [38]. Both approaches indicated that the compounds promptly inhibit CK2 in S and R cells, with similar efficacy, without affecting the amount of CK2. Figure 2 shows the results obtained with CX-4945, in CEM, U2OS, and LAMA84 cells, while in Figure 3 the effects of CX-5011 on CEM cells is shown; similar results were observed in response to treatment of the other cell lines (not shown). As evident in Figure 2B, the expression of CK2 significantly differs in S- and R- CEM cells, as already reported [27], and, in untreated cells (see control lanes, marked as “-” in Figure 2B), it correlates with the phosphorylation level of endogenous substrates Cdc37 Sp13 (phospho-Ser13) and Akt Sp129 (phospho-Ser129), despite the low level of Akt in R-CEM. In all cases, at submicromolar concentrations of the compounds, CK2 activity is reduced by more than 50%, and a parallel dephosphorylation of endogenous substrates occurs. Interestingly, a 6 h treatment is sufficient to inhibit CK2 to almost the maximal level, and longer treatment times only minimally increase the degree of inhibition (Figure 2A). The reduction of Akt Sp129 is also promptly induced (6 h, 0.5 µM CX-4945), while the CK2 target site on Cdc37 seems to be more resistant to dephosphorylation, which is clearly observed only at higher concentrations and longer treatments (Figure 2B). Similar results are observable in response to CX-5011 (not shown and Figure 3 for 24 h treatment).

**Figure 4:**
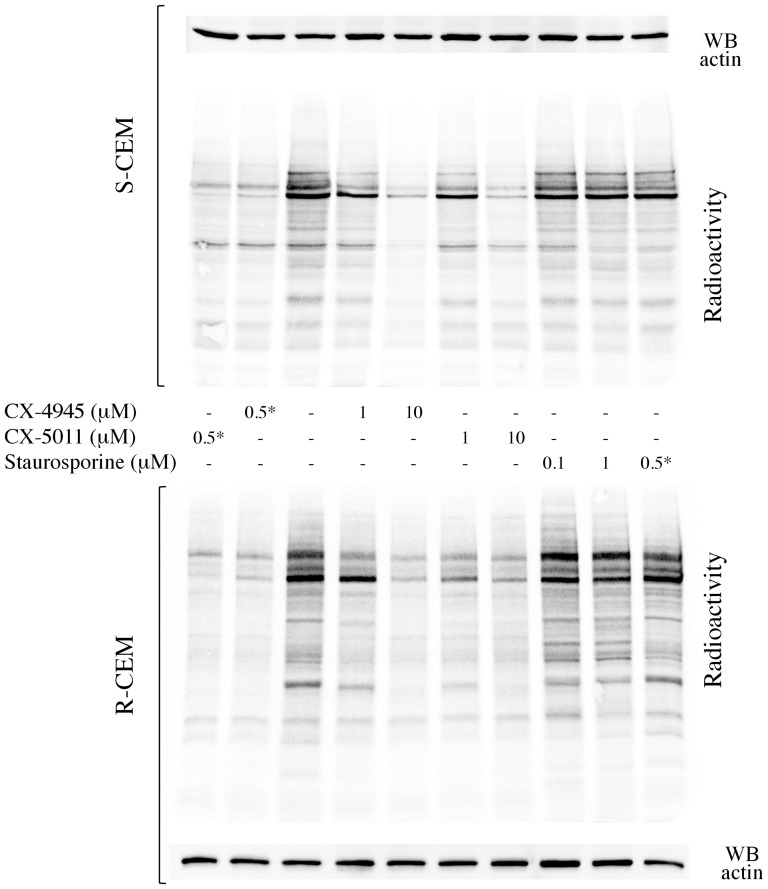
Protein phosphorylation in lysates from cells treated with CX-4945, CX-5011, or staurosporine. S-CEM (upper panel) or R-CEM (lower panel) were treated with the indicated concentrations of the CX inhibitors or staurosporine for 16 h, then lysed. 5 µg of total proteins were incubated with a radioactive phosphorylation mixture, resolved by SDS-PAGE, blotted, and analyzed by digital autoradiography (radioactivity). WB for actin was used as loading control. Asterisk * denotes samples where the inhibitors were added in vitro during the phosphorylation assay (not administrated to the cells). Representative results of five independent experiments are shown.

Inhibition of CK2 was also confirmed by analyzing the radioactive phosphorylation of endogenous proteins in treated cells. Figure 4 shows that several protein bands are less phosphorylated in S- and R-CEM cells treated with CX-4945 or CX-5011 (similarly to what is observable when the compounds are added in vitro, during the phosphorylation assay). To rule out the possibility of a non-specific effect, we treated cells with staurosporine, at concentrations which inhibit the majority of protein kinases but not CK2 [39]. This treatment, while inducing a high degree of cell death (not shown) similarly to what caused by the CK2 inhibitors (see below), it is almost ineffective on endogenous protein phosphorylations; this also suggests that, under the used conditions, CK2 is the major kinase responsible for the observed radioactivity, as expected for a highly expressed, pleiotropic and constitutively active enzyme.

**Figure 5:**
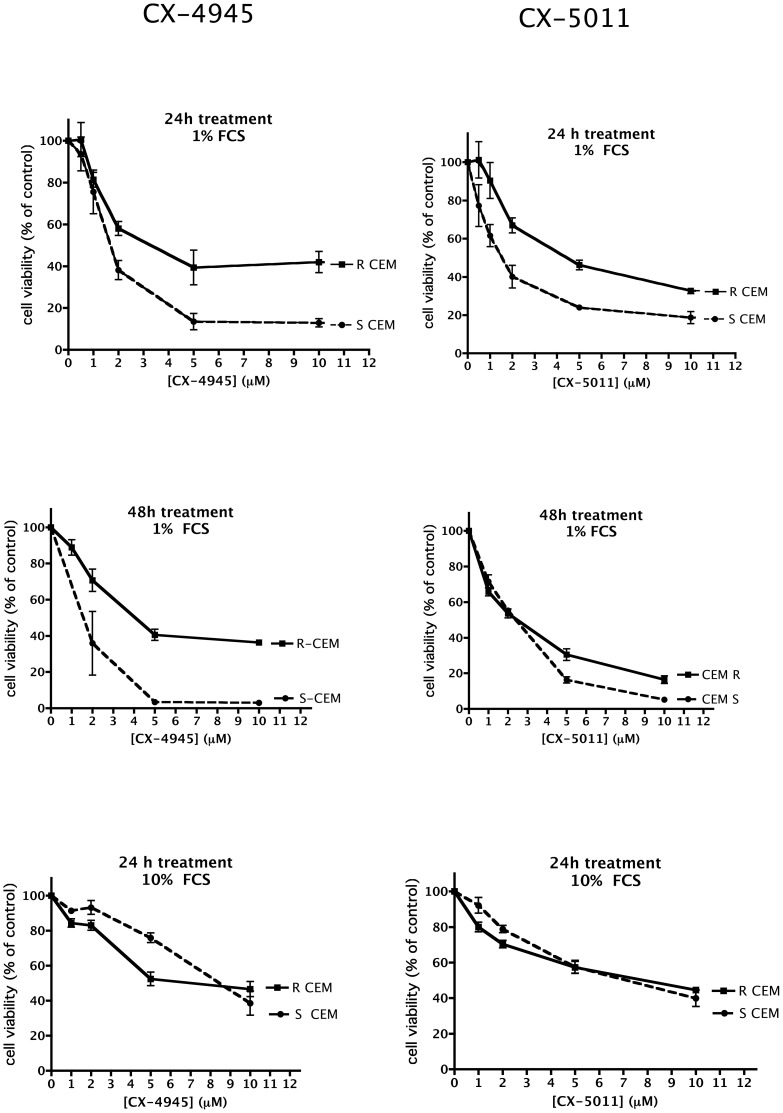
Cell viability of CX-4945 and CX-5011 treated CEM cells. Cells were incubated with increasing concentrations of the CX compounds; the FCS concentration in the medium and the treatment time are indicated on each graph. Cell viability was assessed by the MTT method, assigning 100% value to the vehicle-treated control cells. Mean ± SE values of at least four independent experiments are reported.

**Figure 6:**
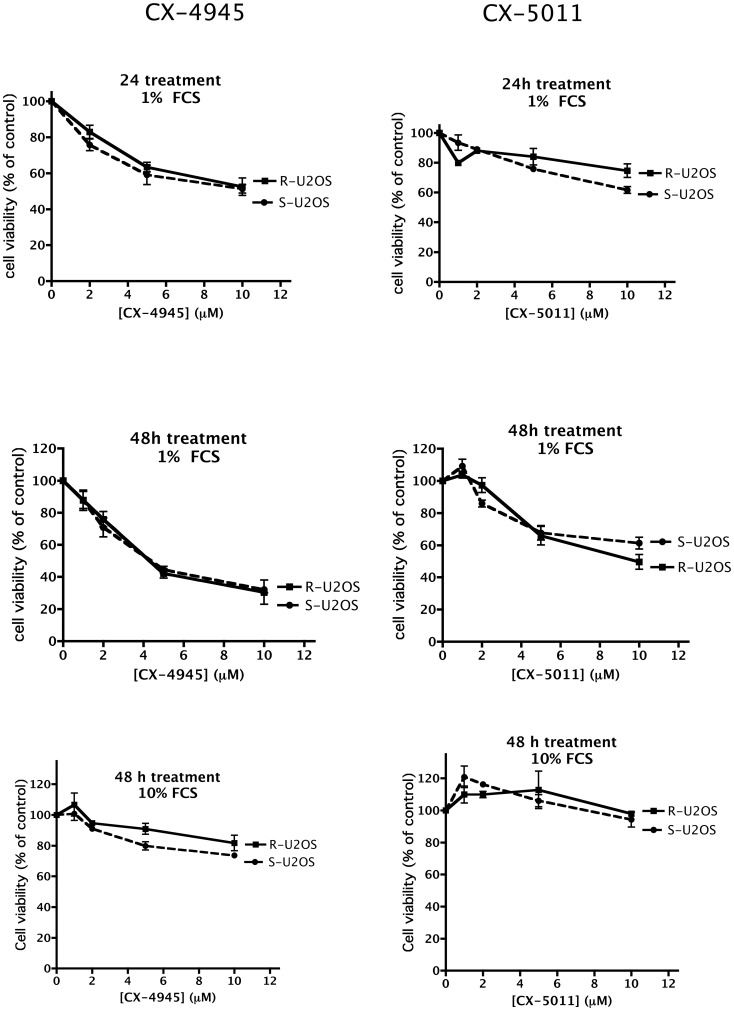
Cell viability of CX-4945 and CX-5011 treated U2OS cells. See legend of Figure 5 for details.

**Figure 7:**
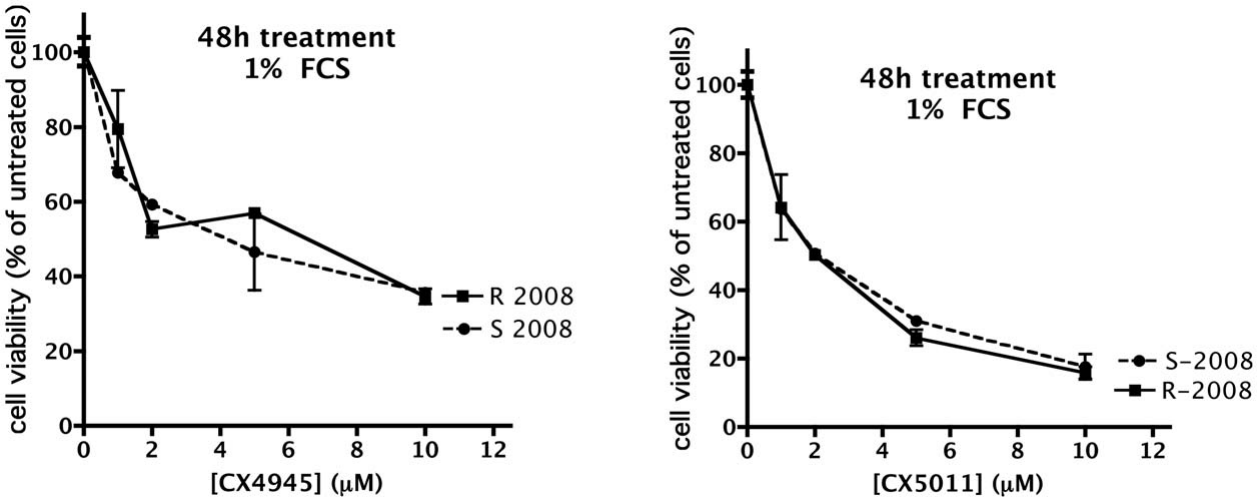
Cell viability of CX-4945 and CX-5011 treated 2008 cells. See legend of Figure 5 for details.

**Figure 8:**
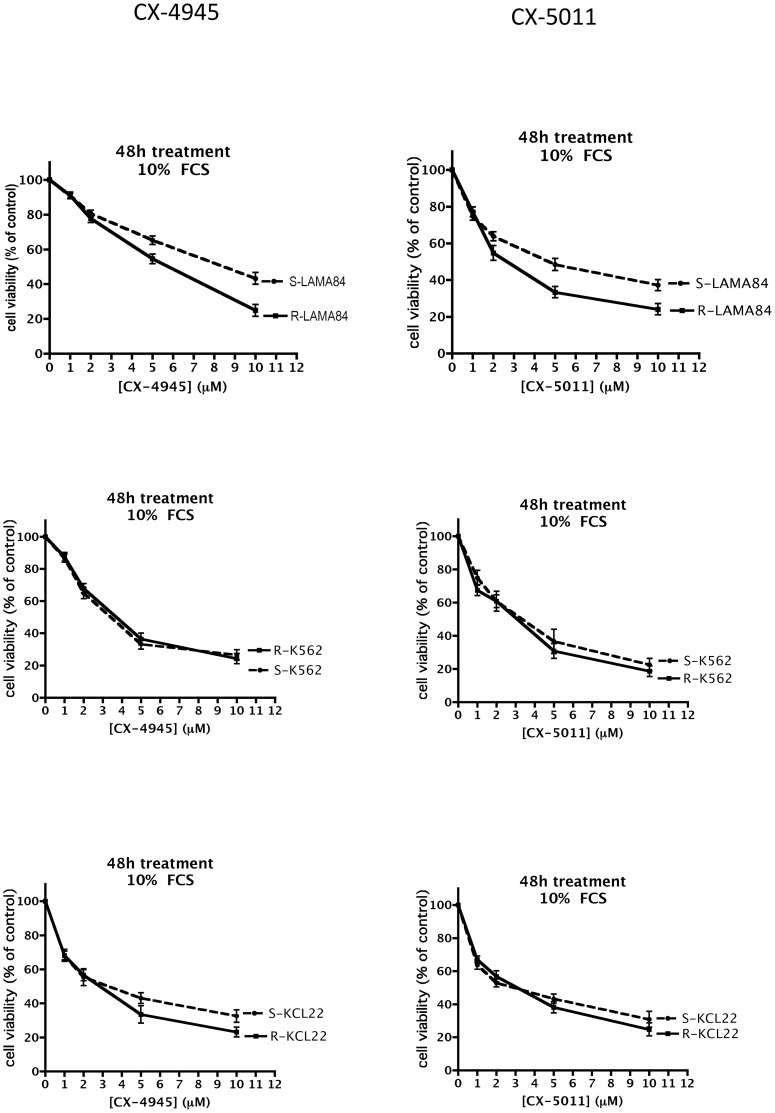
Cell viability of CX-4945 and CX-5011 treated Imatinib-resistant cells. See legend of Figure 5 for details.

To ascertain if CK2 inhibition by CX-4945 and CX-5011 is effective in inducing cell death also in case of drug resistance, we treated cells with increasing concentrations of the compounds, and measured cell viability by the MTT method. Figures 5, 6, 7, 8 show representative results obtained under different time and assay conditions. We found that both inhibitors are able to induce appreciable cell death also in R cells, in a manner quite similar to S cells (at least when 10% FCS is present, see Discussion). In particular, the responsiveness of MDR cells (R-CEM and R-U2OS) indicates that these compounds are not substrate of the Pgp. Table 1 shows the DC_50_ values calculated from the MTT assays shown in Figure 5, 6, 7, 8; the 48 h assays were used for calculation, except for CEM cells in the presence of 10% FCS, where 24 h assays were considered.

**Table 1:** DC_50_ of CX-4945 and CX-5011.

	CEM		CEM*		U2OS		2008		LAMA84		K562		KCL22	
	1% FCS		10% FCS		1% FCS		1% FCS		10% FCS		10% FCS		10% FCS	
	S	R	S	R	S	R	S	R	S	R	S	R	S	R
**CX-4945**	1.92	4.09	8.62	6.50	4.58	4.33	4.87	6.56	8.55	5.79	3.41	3.72	3.30	2.95
**(µM)**	±1.00	±0.27	±0.68	±1.40	±0.44	±0.63	±2.50	±0.48	±1.10	±0.86	±0.55	±0.42	±1.46	±0.89
**CX-5011**	2.40	2.51	7.51	7.59	>10	8.59	2.13	2.01	5.07	2.68	3.58	3.05	3.01	3.10
**(µM)**	±0.07	±0.60	±2.33	±1.35		±0.62	±0.02	±0.21	±1.42	±0.81	±1.18	±0.78	±1.39	±0.86

The values, calculated from 48 h MTT assays reported in Figures 5, 6, 7, 8 (except for CEM* = 24 h treatment), are the mean of 4–6 independent experiments ± Standard Deviation. FCS concentration (v/v) during the treatment is also indicated.

**Figure 9:**
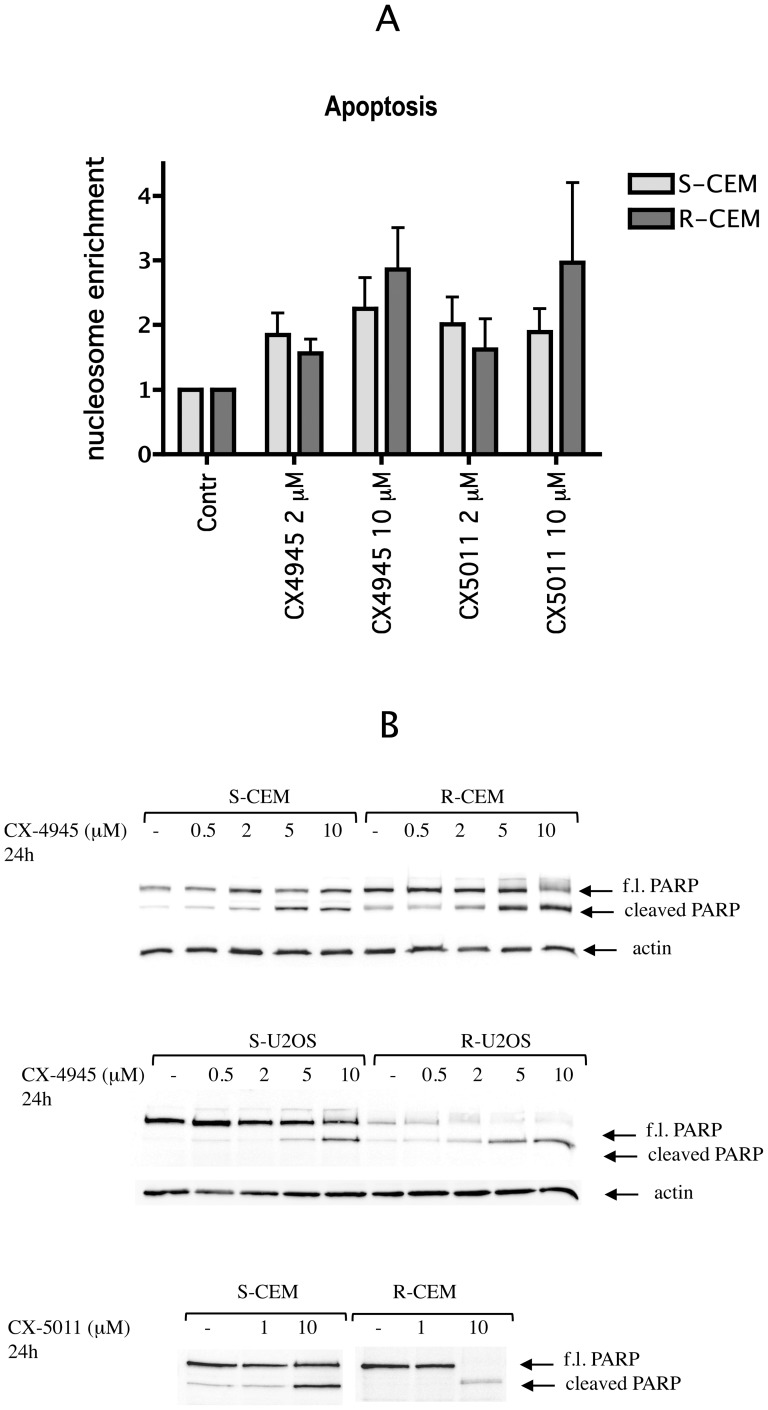
Apoptosis induction by CX-4945 and CX-5011. (A) Apoptosis was assessed by evaluation of nucleosomes present in the cytosol, using the Cell Detection Elisa kit (Roche), after 8 h of CEM cell treatment as indicated. Nucleosome enrichment was calculated from the ratio between the signal in treated and untreated cells; reported values are the means ± SE of four independent experiments. (B) Caspase-dependent PARP cleavage was analyzed by WB on 10 µg proteins of lysate from CEM or U2OS cells treated as indicated. Actin WB was used as loading control. Representative WB of three independent experiments are shown. f.l. PARP: full length PARP.

To understand if cell death induced by CX-4945 and CX-5011 in our cell lines was due to apoptosis, we evaluated the formation of nucleosomes in treated cells; the results, shown in Figure 9 for CEM cells, and confirmed for the other cell lines (not shown), indicated that apoptosis occurs to a similar degree in S and R cells, in response to these CK2 inhibitors. The results are also confirmed by the cleavage of the caspase substrate PARP (Figure 9).

**Figure 10:**
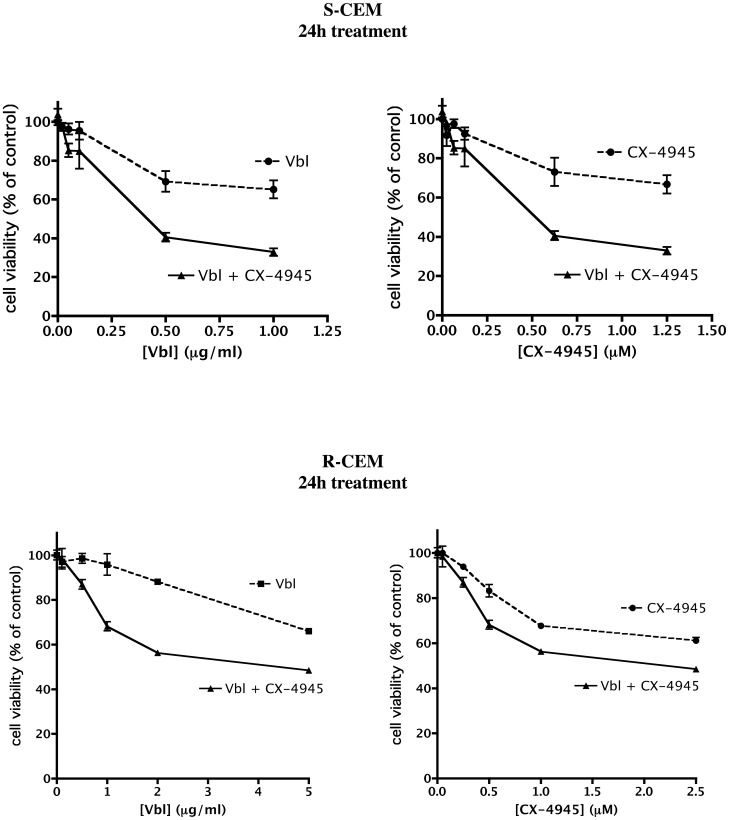
Effect of combined treatment with CX compounds and chemotherapeutic drugs. S-CEM and R-CEM viability was assessed by the MTT method after 24 h treatment with increasing concentrations of both CX-4945 and Vbl, administrated alone or in combination, at fixed ratio (1 µM CX-4945 : 0.8 µg/ml Vbl for S-CEM, 1 µM CX-4945 : 2 µg/ml Vbl for R-CEM, due to their different sensitivity to Vbl). Viability (mean values ± SE of four experiments) was plotted as function of Vbl concentrations (left panels), or CX-4945 concentrations (right panels).

Then we assessed if the treatment with CX compounds can sensitize resistant cells towards conventional antitumor drugs; in particular, we considered the Vbl-resistant R-CEM cells, and we evaluated if the combined treatment with CX-4945 induces a higher degree of cell death in response to Vbl. As shown in Figure 10 (lower panels), very low concentrations of CX-4945 are able to significantly reduce the DC_50_ of Vbl (from 39.4 to 6.2 µg/ml). The calculated Combination Index (CI) is indeed 0.66±0.04, where values <1 indicate synergism [35]. Interestingly, CX-4945 exerts a synergistic effect with Vbl also in S-CEM: as shown in Figure 10, upper panels, the effect of very low concentrations of Vbl on cell viability is increased by the simultaneous administration of the CK2 inhibitor (CI = 0.70±0.08), indicating that the combined treatment can be applied for therapy with significantly lower drug doses. An important synergistic effect is also observable between Imatinib and CX-4945 on Imatinib-resistant cells (manuscript in preparation).

**Figure 11:**
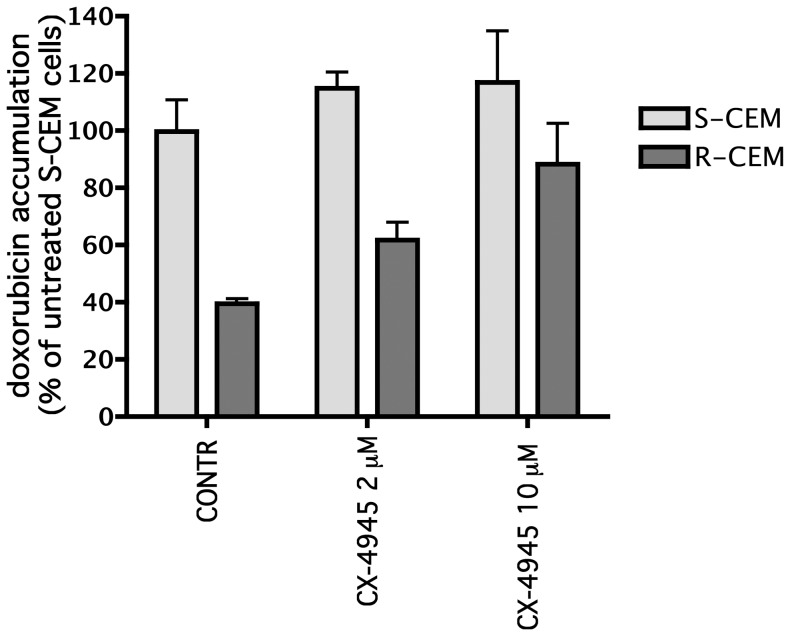
Effect of CX-4945 on doxorubicin accumulation. CEM cells were preincubated for 30 min with vehicle (Contr) or CX-4945 at the indicated concentrations, then further incubated for 30 min with 25 µM doxorubicin, whose amount, detected fluorimetrically as described in Materials and Methods, is reported as percentage of the value found in control S-CEM (mean ± SE of three independent experiments).

Since the extrusion pumps expressed in MDR cells are phosphorylated by CK2 [25], [26], and an activation effect has been clearly demonstrated, at least in the case of MRP1 [26], we finally assessed whether a short treatment with CX-4945 allows a higher accumulation of conventional drugs inside resistant cells. To this purpose, exploiting the fluorescence of doxorubicin, we measured its uptake in CEM cells pre-treated with CX-4945, compared to untreated cells (Figure 11), and we found that the inhibitor markedly increases the amount of drug retained inside R-CEM cells, while it is almost ineffective in S-CEM cells.

## Discussion

Although CK2 is not considered a direct cause of cancer and cannot be strictly defined as an oncogene, its high abundance in cancer cells is indicative of its importance in tumorigenesis. We have previously hypothesized [40] that, whenever, for any reason, a cell displays a higher level of CK2, that cell will have a survival advantage over the other cells, and will be selected to proliferate under the pressure represented by treatment with pro-apoptotic drugs. CK2 is thus expected to play a major role in the apoptosis resistant phenotype, as also suggested by previous studies [27], [41]. Moreover, since CK2 expression is not linked to specific types of cancers, its targeting could be a successful strategy, because of its very general applicability and widespread effects.

Importantly, cancer cells are expected to be more sensitive to CK2 inhibition than normal cells, since they are addicted to CK2, strongly relying on it for their survival [6]. Accordingly, the CK2 inhibitors have proved to be more effective in tumor cell lines than in normal ones [17], [42], and, on these bases, CX-4945 has entered clinical trials, with promising initial results. Here we demonstrate that the CX inhibitors are also able to overcome the problem of resistance to apoptosis, since they are similarly effective in resistant cells (R) and their normally sensitive counterparts (S). Interestingly, our R cell lines display different kind of apoptosis resistance: R-2008 cells are resistant to cisplatinum; R-LAMA84, R-KCL22 and R-K562 are resistant to Imatinib, while R-CEM and R-U2OS are MDR cells, expressing the Pgp pump. Therefore, the first outcome of our data is that CK2 inhibition has a general effect on resistant cells, by reducing the efficacy of cellular equipment to escape apoptosis; secondly, we can conclude that CX-4945 and CX-5011 inhibitors are not recognized by the Pgp, since their effects are visible in cells expressing this MDR pump.

An observation from our results is that the CX compounds, especially CX-5011, seem to be less effective with U20S cells than with the other cell lines (compare Figure 6 with Figures 5, 7 and 8); the reasons are presently unknown, however they are not related to the MDR phenomenon, being the response very similar in the S and R variants of this cell line.

It can also be observed that the inhibitors are slightly less effective in R-CEM than in S-CEM; however the difference between the two variants is detectable on cell viability and not on endogenous CK2 inhibition, and is abrogated when cell viability assays are performed in the presence of 10% instead of 1% FCS (Figure 5). Although we do not know the exact reason of the different results observed at 10% or 1% FCS, our interpretation is that, at quite critical conditions (such as 1% FCS), the anti-apoptotic machinery of R-CEM is more effective than that of S-CEM in protecting cells from the stress represented by CK2 inhibition; on the contrary, under fully healthy conditions (10% FCS), the two cell variants are equally equipped to counteract apoptosis. In any case, these findings suggest that any observed difference is not due to extrusion of the inhibitors by the Pgp, as also confirmed by the results obtained with other Pgp-expressing cells (compare S-U2OS and R-U2OS, Figure 6).

We have previously found that R-CEM display a higher level of the CK2 catalytic subunit compared to S-CEM [27], and this is confirmed by the results here shown in Figure 2B, where it is also evident that the phosphorylation state of CK2-dependent sites (particularly of Akt Sp129) in R-CEM is higher than in S-CEM. Interestingly, despite the different endogenous CK2 activity, the degree of inhibition induced by CX-4945 and CX-5011 is very similar in S- and R-CEM cells (Figure 2A).

While it is obviously confirmed that CK2 blockade causes apoptosis, an interesting observation emerging from our results is that cell death is appreciable only when the degree of CK2 inhibition induced by the CX compounds is sufficiently high to ensure a dephosphorylated state of major substrates. In fact we found that, while the endogenous CK2 activity towards a peptide substrate is already halved in cell treated with <0.5 µM inhibitors (see for example 24 h treatment in CEM cells, Figure 2), significantly higher concentrations are required to induce 50% cell death (see Figure 5). However, if we consider the phosphorylation states of the CK2 sites analyzed by phospho-specific antibodies, we observe that while Akt Sp129 is promptly reduced, Cdc37 Sp13 phosphorylation is much more stable. Of course, extending our considerations to the multitude of CK2 substrates, we can presume that each one has its own susceptibility to CK2 inhibition, that will mainly depend on the turnover of its phosphorylation state; since this is obviously the result of the balance between kinase and phosphatase activity, there will be a variability depending on cell type and conditions. We cannot exclude that the dephosphorylation of one or few specific CK2 substrates is required before cell death occurs; alternatively, we can assume that only a massive dephosphorylation of CK2 substrates will produce cell death, whose extent, therefore, does not necessarily correlate with the decrease of CK2 catalytic activity.

Another important outcome of our data is that CX-4945 can be useful to sensitize resistant cells to conventional chemotherapeutic drugs. It has already been reported that CX-4945 augments the anti-tumor efficacy of gemcitabine and cisplatin on ovarian cancers [43]. The association of CX-4945 with Erlotinib, an EGF-receptor inhibitor, has also been proposed [44]; in this case, interestingly, the enhanced killing effect of the combined treatment is accompanied by a reduction of the PI3K-Akt-mTOR pathway, with the complete abrogation of Akt phosphorylation. Here we extend the drug-combination experiments to MDR cells, showing that R-CEM are six fold more sensitive to Vbl when simultaneously treated with sub-lethal doses of CX-4945, compared to when treated with Vbl alone. Moreover, we also found that the CK2 inhibition by CX-4945 allows an increased accumulation of doxorubicin in R-CEM (Figure 11), most probably blocking the positive effect that CK2 exerts on Pgp. As expected, the drug accumulation is unaffected by CX-4945 in S-CEM, not expressing Pgp; since the synergism between CX-4945 and chemotherapeutic drug is instead observable also in S-CEM (Figure 10), we have to assume that additional mechanisms of increased cell death, other than the effect on the Pgp pump, are induced by the combined treatment.

A parallel study has been undertaken for the synergistic effect of Imatinib and CX-4945 in Imatinib-resistant LAMA84 cell line (manuscript in preparation).

In summary, our results show that CX-4945 and CX-5011 are internalized in resistant cells, inhibit endogenous CK2, and, alone or in combination with other drugs, induce significant cell death. We suggest that they can be seriously considered as a valid therapeutic strategy also in case of pharmacological resistance occurrence.
